# A Deadly Embrace: Hemagglutination Mediated by SARS-CoV-2 Spike Protein at Its 22 N-Glycosylation Sites, Red Blood Cell Surface Sialoglycoproteins, and Antibody

**DOI:** 10.3390/ijms23052558

**Published:** 2022-02-25

**Authors:** David E. Scheim

**Affiliations:** US Public Health Service, Commissioned Officer, Inactive Reserve, Blacksburg, VA 24060, USA; dscheim@alum.mit.edu

**Keywords:** SARS-CoV-2, spike protein, COVID-19, betacoronavirus, sialic acid, glycophorin A, CD147, hemagglutination, hemagglutinin esterase, α5-N-acetylneuraminic acid (Neu5Ac)

## Abstract

Rouleaux (stacked clumps) of red blood cells (RBCs) observed in the blood of COVID-19 patients in three studies call attention to the properties of several enveloped virus strains dating back to seminal findings of the 1940s. For COVID-19, key such properties are: (1) SARS-CoV-2 binds to RBCs in vitro and also in the blood of COVID-19 patients; (2) although ACE2 is its target for viral fusion and replication, SARS-CoV-2 initially attaches to sialic acid (SA) terminal moieties on host cell membranes via glycans on its spike protein; (3) certain enveloped viruses express hemagglutinin esterase (HE), an enzyme that releases these glycan-mediated bindings to host cells, which is expressed among betacoronaviruses in the common cold strains but not the virulent strains, SARS-CoV, SARS-CoV-2 and MERS. The arrangement and chemical composition of the glycans at the 22 N-glycosylation sites of SARS-CoV-2 spike protein and those at the sialoglycoprotein coating of RBCs allow exploration of specifics as to how virally induced RBC clumping may form. The in vitro and clinical testing of these possibilities can be sharpened by the incorporation of an existing anti-COVID-19 therapeutic that has been found in silico to competitively bind to multiple glycans on SARS-CoV-2 spike protein.

## 1. Introduction

Although COVID-19 typically gains infectious penetration in the respiratory epithelium [1,2,3], vascular damage is frequently observed in lungs and other organ systems of COVID-19 patients, with morbidities such as intravascular clotting, microvascular occlusion and peripheral ischemia [4,5,6,7,8,9,10,11,12,13,14,15,16,17]. Histological studies from COVID-19 patients have found extensively damaged endothelium of pulmonary capillaries adjoining relatively intact alveoli [18,19], corresponding to hypoxemia with normal breathing mechanics observed in patients with this viral disease [10,16,17,18,20,21]. One clinical reviewer characterized COVID-19 as “a systemic disease that primarily injures the vascular endothelium” [21].

**Figure 1:**
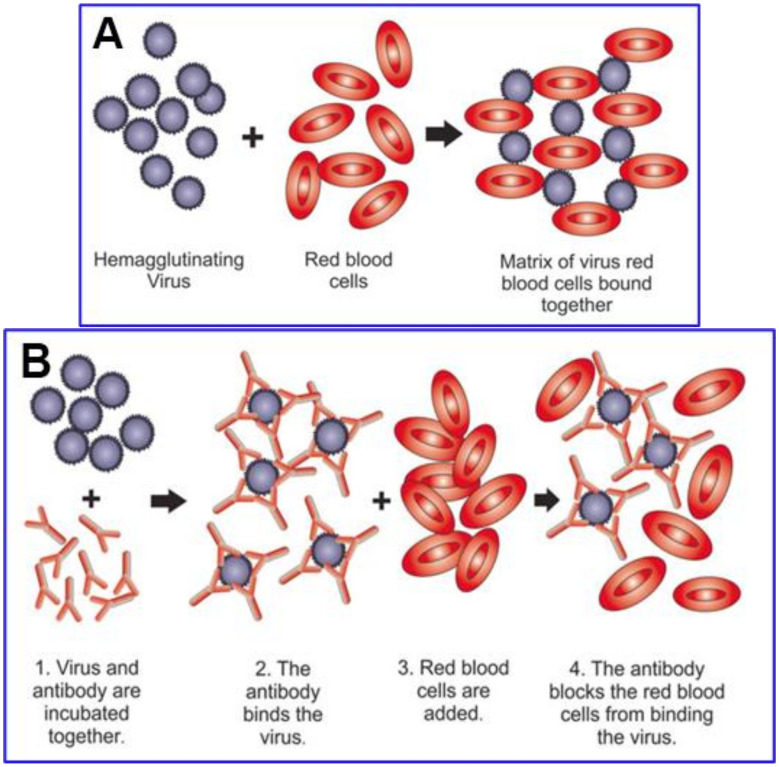
Schematic of hemagglutination (**A**) and hemagglutination inhibition (**B**), both of which occur either in vitro in their respective assays or in vivo. Reproduced with permission from Springer Nature ((**A**) Killian, 2014 [57]; (**B**) Pedersen, 2014 [60]). (**B**) depicts blockage of hemagglutination by an antibody to the virus.

A framework for studying the vascular occlusive morbidities characteristic of COVID-19 is provided by viral properties dating back to classic experiments of the 1940s, as reviewed [22]. Viruses fuse and then replicate via host cell receptors specific to the viral strain, which is ACE2 for SARS-CoV-2 [23,24]. However, many enveloped viral strains, including coronaviruses, initially attach to host cell membranes via glycoconjugate molecules, including those tipped with sialic acid (SA) [25,26,27,28,29,30]. SA is densely distributed on red blood cells (RBCs) as terminal residues of its surface sialoglycoprotein, glycophorin A (GPA), and of its CD147 transmembrane receptors [31,32,33,34]. Through viral bindings to SA surface moieties to be considered in detail below, SARS-CoV-2 agglutinates RBCs, as established in vitro [35], with such bindings also demonstrated clinically [36]. Hemagglutination also occurs for several other viral strains including other coronaviruses [35,37,38,39,40,41,42,43,44,45,46,47,48], as demonstrated in the classic viral hemagglutination assay. In that assay, developed in the 1940s [38,49,50,51] and refined later by Jonas Salk [52,53,54,55,56], virus particles are mixed with RBCs to form a hemagglutinated sheet [40,57,58,59]. However, for viruses that express enzymes that cleave SA, that sheet subsequently collapses as these enzymes disintegrate SA-binding sites on the RBCs [38,58,60,61]. Figure 1 shows a schematic of virally mediated hemagglutination and of its inhibition through an agent that competitively binds to attachment sites on the virus, in this case an antiviral antibody.

### 1.1. Binding of Viruses to SA and Host Decoy Defense

For viruses that bind to SA, including SARS-CoV-2 as noted above, such glycan bindings play a key role in viral infectivity, as SA typically functions as an initial point of attachment to host cells [25,30,44,48,62,63,64,65,66,67,68,69,70]. The human host, reciprocally, protects against attachment of virions to host cell infectious targets by presenting SA on a set of decoys, including RBCs as well as platelets and leukocytes [22,31,71,72,73]. In particular, SA moieties are densely distributed on the RBC surface (35 million per cell [33]), mainly as terminal residues of both the CD147 receptor [22] and GPA [31,74], with GPA serving no known physiological role other than as a decoy for pathogens [31,72,73,75]. RBCs perform an active pathogen clearance role, attaching to microorganisms and then delivering them to leukocytes or conveying them to macrophages in the liver or spleen for phagocytosis [31,71,73,76]. With bacteria, this clearance by RBCs requires both antibody and complement [77,78]. Viruses, however, are snagged directly by RBCs, and viral–RBC binding is inhibited by antiviral antibodies [22]. RBCs are well suited for this immune defense role, having no nucleus or other infrastructure to perform viral replication and being expendable, numbering 20–30 trillion per human host [72].

### 1.2. Cleavage of Viral–SA Binding via Hemagglutinin Esterase (HE), an Enzyme Expressed by the Common Cold Betacoronavirus Strains but Not by SARS-CoV, SARS-CoV-2 and MERS

Hemagglutinin esterase (HE) is an SA-cleaving enzyme deployed by certain viral strains, including some betacoronaviruses. HE increases the infectivity of these viral strains by expediting the release of replicated virions from sialoside-binding sites on host cells and by restricting viral snagging on such binding sites of non-infectious targets, including mucins, blood cells and plasma proteins [22]. Viral HE can both bond and cleave host cell sialoside-binding sites [79,80]; however, for betacoronaviruses, even for those expressing HE, attachments to host cell sialoside-binding sites are effected mainly through glycans at 22 N-glycosylation sites on viral spike protein S1, in particular, eight of those clustered on the N-terminal domain (NTD) [44,81,82,83,84,85,86,87]. Genomic analysis of the five strains of human betacoronaviruses reveals expression of the HE enzyme in those that cause the common cold, OC43 and HKU1, but not in the three deadly strains, SARS-CoV, SARS-CoV-2 and MERS [88,89,90,91,92].

### 1.3. Multivalent Binding

To adjust for a missing release capability in the three betacoronavirus strains lacking HE, SA-binding affinity is diminished, while multiplicity of binding provides robust variations in attachment strength [44,48,62,63,93,94]. These viral bonds to SA and other glycans have weak individual affinities but greatly increased collective strength [25,30,44,48,63,66,93,94,95,96]. The dissociation constant Kd for a multivalent attachment of viral spike protein to SA increases exponentially (in absolute value) as a function of the number of bonds [97,98]. For example, the Kd of an individual bond of viral spike protein to a host sialoside-binding site is in the low millimolar range [63,93,99,100], but the Kd value would be in the nanomolar range for a triple bond [97]. A single sialoside bond for a virus particle initially attaching to a cell would provide only a tentative foothold [25,30,44,63,101,102], allowing migration to an ACE2 receptor for fusion and replication.

## 2. Properties of SARS-CoV-2 Related to Its Array of Spike Glycoprotein Glycans

### 2.1. SARS-CoV-2 Binds to SA and CD147

Because bindings from viral spike protein to SA are generally weak when univalent, several coronavirus strains require a nanoarray experimental detection methodology to register viral attachment to hosts [22,48,61,62]. Using such a methodology, a nanoparticle array bearing SA derivatives, the binding of both SARS-CoV-2 spike protein and pseudovirus was demonstrated [94]. This study’s detection system is adaptable to mass COVID-19 screening, with a 5 nM concentration threshold for detection of viral spike protein. Another attachment point on host cells for the SARS-CoV-2 virus is the CD147 transmembrane receptor, which contains SA at its terminal domains [34,103]. Binding of SARS-CoV-2 spike protein to CD147 was shown by enzyme-linked immunosorbent assay (ELISA), surface plasmon resonance (SPR) and co-immunoprecipitation (Co-IP) assays, and colocalization of CD147 and viral spike protein was revealed on infected Vero E6 cells [104]. Meplazumab, a humanized anti-CD147 antibody, was found to inhibit viral proliferation in vitro and to provide significant clinical benefits in a small study of its use for COVID-19 treatment [105].

### 2.2. SARS-CoV-2 Attaches to RBCs, Other Blood Cells and Endothelial Cells

Several of the glycans at the 22 N-glycosylation sites and both O-linked glycans on SARS-CoV-2 spike protein (as depicted in Figure 3) are capped with SA monosaccharides of the same type that are densely distributed on human RBCs at the tips of GPA molecules, as will be considered in some detail below. These matching glycans enable SARS-CoV-2 to hemagglutinate when mixed with human RBCs, as indeed demonstrated using the hemadsorption assay [35] (similar to the hemagglutination assay [106,107]). Hemagglutination occurs more generally in eight families of viruses, including other coronaviruses [35,37,38,39,40,41,42,43,44,45,46,47,48]. Attachment of SARS-CoV-2 spike protein to RBCs was demonstrated directly through immunofluorescence analysis of RBCs from the blood of nine hospitalized COVID-19 patients [36]. The mean percentage of RBCs having SARS-CoV-2 spike protein punctae was 41% at day 0 of hospital admission, with values ranging from 0% for one patient and 18% for two patients to 79% for another patient. This mean percentage increased to 44% at day 7 after hospital admission.

Like RBCs, several other cell types express surface SA glycoconjugates and can thus also attach to SARS-CoV-2 virus particles. SA and SA-tipped CD147 are expressed on endothelial cells of blood vessel linings (luminal surface), platelets, lymphocytes, macrophages, and other types of white blood cells [22]. The potential for pathogen attachments to SA and CD147 that impede vascular blood flow is indicated in another disease, severe malaria, in which the malaria parasite attaches to SA-binding sites on an RBC [73,108,109] and penetrates the RBC through the latter’s CD147 receptors [110,111]. Clumps develop between infected and uninfected RBCs, often including platelets, which, along with endothelial cytoadhesion by infected RBCs, cause vascular occlusion, the key morbidity of severe malaria [22].

**Figure 2:**
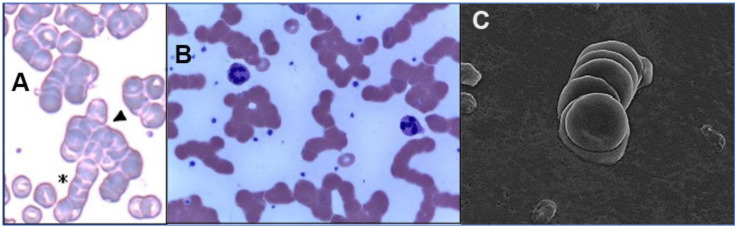
Images of RBC rouleaux (clumps) from the blood of COVID-19 patients, obtained using light ((**A**) [112], (**B**) [113]) and electron microscopy ((**C**) [114]). The first study (**A**) found huge rouleaux formation by RBCs in 85% of COVID-19 patients studied [112]; the second (**B**) found these in 33% of patients [113]; and the third (**C**) found these prevalent in its series of 31 patients, all with mild COVID-19 [114]. Reproduced with permission from (**A**) SIMTIPRO Srl; (**B**) CC-BY 4.0; (**C**) Georg Thieme Verlag KG.

The interlaced attachments of RBCs with SARS-CoV-2 virions as observed in vitro in the hemadsorption assay [35] may well define the mechanism by which clumps (rouleaux) of RBCs form in the blood of COVID-19 patients [112,113,114], as shown in Figure 2. These clumps would present vascular obstructive potential, given that an RBC of average disk diameter 8 µm [115,116] traverses through an alveolar capillary of smaller average cross-sectional diameter [117], achieved only by a distortion of the RBC’s shape to press against the capillary wall [116,118]. Such RBC clumps could be a prime cause of the microvascular occlusion which, as noted above, is characteristic of COVID-19. These clumps could contribute as well to microvascular occlusion in larger capillaries, of cross-sectional diameter up to 20 µm [115,119], elsewhere in the body. Vascular occlusion would be promoted even though such stacked RBC clumps (rouleaux) typically dynamically aggregate and disaggregate [120,121,122], as do RBC aggregates that can form in the absence of pathogens, promoted by macromolecules in plasma under conditions of low blood shear rates [123,124]. Formation of RBC rouleaux would increase blood viscosity [121,122], impeding blood flow, especially in the small-diameter pulmonary capillaries, which would cascade as reduction in both flow velocity and associated shear forces would in turn tend to favor aggregation vs. disaggregation and further occlude flow [121].

The abundant distribution of SA-tipped CD147 on endothelial cells of blood vessel linings, with 28,000 CD147 receptors (vs. 175 ACE2 receptors) per endothelial cell [125], may be key to the attachments of SARS-CoV-2 to endothelium and the ensuing damage that has been widely observed in COVID-19 patients [15,18,19,126,127,128]. Damage to endothelium caused by SARS-CoV-2 spike protein in the absence of whole virus was demonstrated both in vitro and in vivo in three studies [129,130,131], and presence of isolated SARS-CoV-2 spike protein on endothelial cells was also observed clinically [130,132,133,134,135]. Additionally, one study of 31 hospitalized patients with mild to moderate COVID-19 found that serum levels of circulating endothelial cells (CECs), as determined by different measures, were up to 100-fold the levels for matched controls, and that these CECs from the COVID-19 patients typically each had several holes in their membranes approximately the size of SARS-CoV-2 viral capsid (the viral envelope) [114].

### 2.3. The Glycan Distribution and Composition at the 22 N-Glycosylation Sites of SARS-CoV-2 Spike Protein

**Figure 3:**
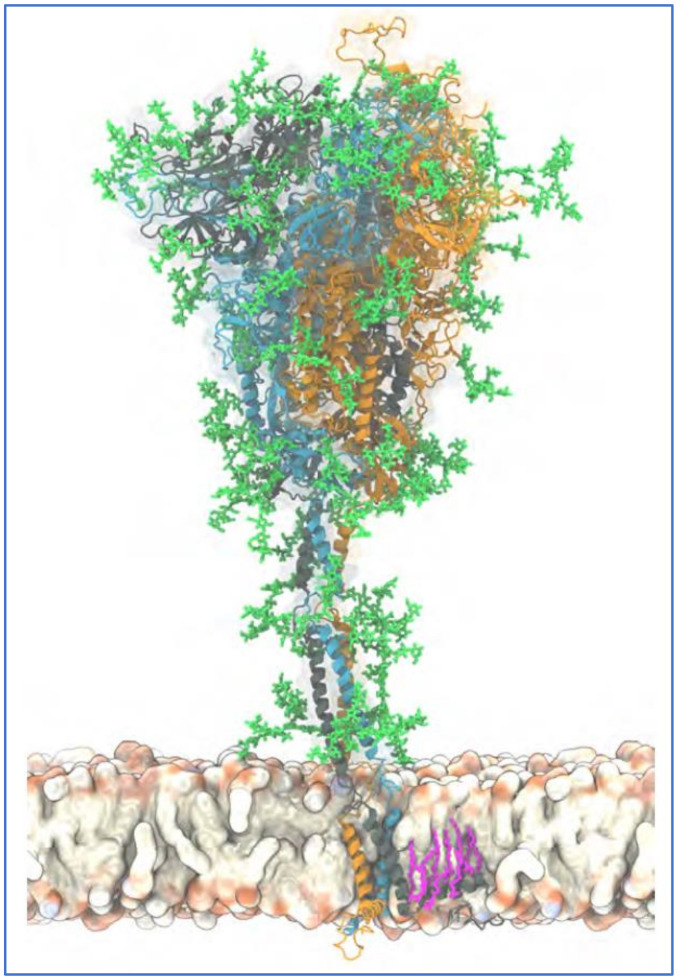
Atomistic model of the full-length trimeric S protein of SARS-CoV-2 shown in cartoon representation. Reproduced from Sikora et al., 2021 [140] (CC-BY 4.0). The three monomeric chains are differentiated by color. Palmitoylated cysteine residues are shown in pink licorice (only one chain shown for clarity), anchored into the viral envelope. Glycans are shown in green licorice representation. A 20 s movie showing a 600 ns atomistic molecular dynamics simulation trajectory of four S proteins embedded in a viral membrane is also provided at this source [140].

To explore key characteristics of glycan-mediated SARS-CoV-2 spike protein binding to host cells, it is helpful to consider the specific distribution and composition of the glycans at its glycosylation sites. SARS-CoV-2 spike glycoprotein is a trimer with a central helical stalk embedded in the viral envelope at its C-terminal end. The stalk consists of three joined S2 subunits each capped with an S1 subunit head spreading out in a mushroom-like shape [136,137,138,139]. An atomistic model of a full-length trimeric SARS-CoV-2 spike protein with its attached glycans, its C-terminal (stalk) end embedded in the viral envelope, is shown in Figure 3. 

**Figure 4:**
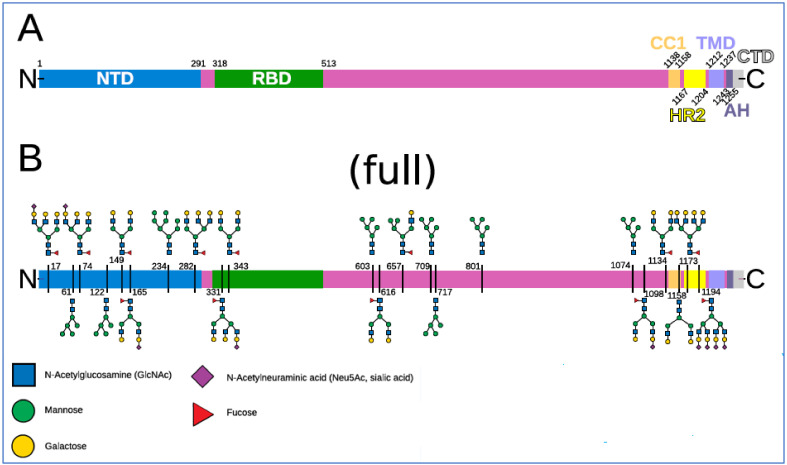
Spike domains and glycosylation. Reproduced from Sikora et al., 2021 [140] (CC-BY 4.0). (**A**) Domains of S (SARS-CoV-2 spike protein). (**B**) Glycosylation pattern of S. Sequons are indicated with the respective glycans in a schematic representation for a fully glycosylated system (“full”). A key to the monosaccharides represented is shown at the bottom.

A map of the 22 N-linked glycans on each of a spike’s three monomers is shown in Figure 4. In addition, two O-linked glycosylation sites, S325 and T323, were identified for each spike monomer, both on S1 RBD [86], and both containing SA terminal monosaccharides [141]. Each SARS-CoV-2 virion has a diameter, excluding spikes, of approximately 100 nm, with the number of spikes estimated at up to 65 per virion, these spikes having a length of approximately 20 nm [142,143,144,145].

The NTD on SARS-CoV-2 spike protein S1, at its N-terminal end, is a focal region for the spike’s glycans—eight of the spike’s 22 N-glycosylation sites are located there [84,85,86]. The NTD is accordingly the typical point of initial viral attachments to glycoconjugate binding sites on host cells [44,81,82,83,84,85,86,87]. After initial attachment, viral fusion to a host cell begins with linkage of the spike’s receptor-binding domain (RBD), situated just below NTD on spike S1, to an ACE2 receptor on the host cell membrane. The S2 stalk then becomes engaged and viral replication proceeds [47,138,146]. The RBD, one on each of a spike’s three monomers, constantly switches between open (“up”) and closed (“down”) configurations, the former enabling both ACE2 binding and immune surveillance, the latter blocking both of those functions [136,147].

Our focus now shifts to specifics of virally-mediated clumping of RBCs, notwithstanding the importance of inflammatory processes and endothelial damage in triggering and exacerbating the morbidities of COVID-19, especially in its critical phase. Additionally, platelets, the second most copious blood component [147], serve a pathogen clearance role like that of RBCs [148,149,150], and like RBCs have abundant CD147 and SA surface molecules [151,152,153,154,155,156,157,158]. Platelets can adhere to viruses, RBCs and endothelial cells, especially under inflammatory conditions [16,22,149], and are often enmeshed in clumps that develop in severe malaria between infected and healthy RBCs [159,160,161,162]. Virally induced clumping of RBCs, however, is of particular interest for these reasons. First, as noted above, this clumping alone could limit the efficiency of blood oxygenation in the pulmonary capillaries. Second, such clump formation is directly testable both by examination of the blood of COVID-19 patients and by mixing spike protein with RBCs in vitro. Third, as will be detailed, if such virally induced RBC clumping is confirmed, an existing drug that has been found in silico to bind to glycan sites on both spike protein and host cells can be tested in vitro and clinically for inhibition of virally mediated RBC clumping, in conjunction with an anti-COVID-19 therapeutic benefit.

Of the 22 N-linked glycosylation sites at each of the three monomers of SARS-CoV-2 spike protein, eight are located on NTD as noted above, two on RBD, three others elsewhere on S1, and the other nine on S2 [84,85,86,87]. N1194 is the closest N-glycosylation site from the C-terminal domain, the end of the spike attached to the virion. In different studies, glycans have been identified as populating between 17 and 21 of these 22 N-glycosylation sites [86,87,163,164]. One study found that ten of these 22 sites have terminal SA moieties, in particular of α5-*N*-acetylneuraminic acid (Neu5Ac), the predominant type of SA found in human cells [25,30]. The terminal monosaccharides on SARS-CoV-2 spike N-glycans other than SA are galactose, mannose, fucose, *N*-acetylglucosamine (GlcNAc) and/or *N*-acetylgalactosamine (GalNAc) [84,87]. As noted above, two SA-tipped O-linked glycans sites have been identified as well, both on S1 RBD [86,141]. Spike protein S1 at its NTD domain was found to bind strongly, in particular to Neu5Ac [94], the type of SA at SARS-CoV-2 N-glycosylation sites and predominant in human cells.

**Figure 5:**
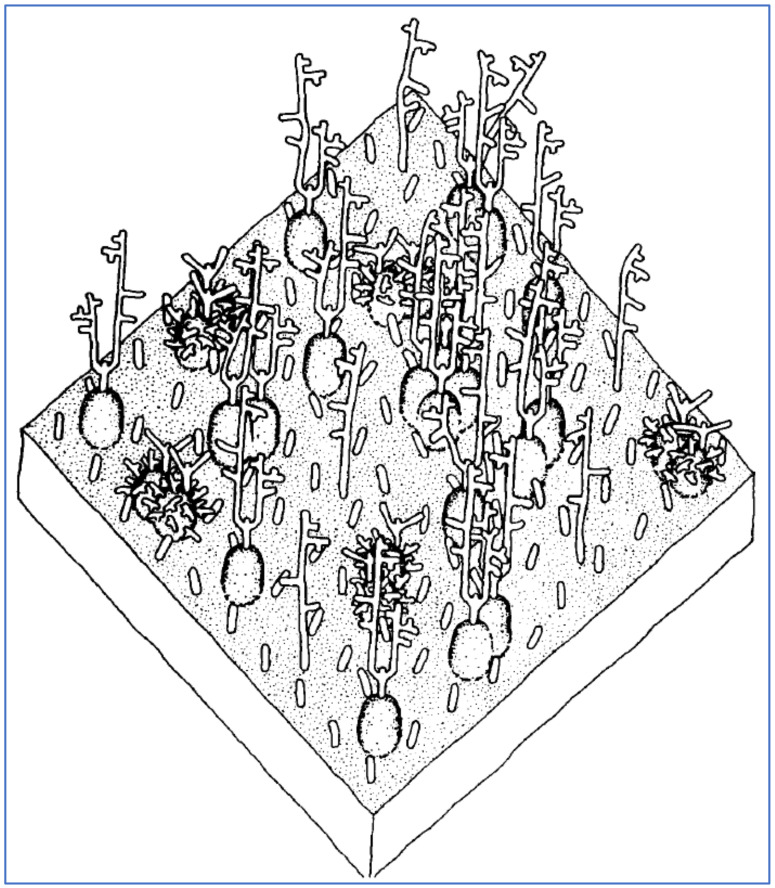
A representation of a 350 × 350 Angstrom area of the RBC surface depicting its sialoglycoprotein coating, consisting of GPA molecules, extending approximately 5 nm from the RBC cell membrane, plus other smaller glycoprotein molecules interspersed. Reproduced with permission from Elsevier (Viitala, 1985 [75]).

GPA, the major sialoglycoprotein in human RBCs, is of central interest in the attachment of SARS-CoV-2 to RBCs, as observed in vitro [35] and on RBCs of COVID-19 patients [36] as noted above. GPA populates human RBCs at approximately one million molecules per cell and contains most of the SA (of type Neu5Ac) on them [31,33,74,165,166]. GPA molecules have the shapes of strands that are anchored approximately 14 nm apart on the RBC plasma membrane, each extending outwards 5 nm [75]. GPA constitutes the bulk of the RBC’s sialoglycoprotein coating, thus determining its 5 nm thickness [75,167,168], and accounts for most of its negative charge [31,169]. Electrostatic repulsion imposes a minimum distance of approximately 8 nm between the outer boundaries of those sialoglycoprotein coatings of adjoining RBCs in a static suspension, but a smaller separation can be achieved when additional forces are pushing these RBCs together [168,170]. SA in its predominant human form, Neu5Ac, is the most common terminal residue of GPA, with its other terminal monosaccharides matching those on SARS-CoV-2 spike N-glycans as noted above: galactose, mannose, fucose, *N*-acetylglucosamine (GlcNAc) and *N*-acetylgalactosamine (GalNAc) [74,75,166]. A representation of a portion of an RBC membrane with strands of GPA and with other glycoproteins interspersed is shown in Figure 5.

## 3. Potential Scenarios for Virally Induced RBC Clumping and Damage to Endothelial Cells

With this background in the underlying biochemistry, specific scenarios can be considered as to how the SARS-CoV-2 virus would interact with RBCs to yield clumps, as manifested in the rouleaux found in COVID-19 patients (Figure 2). First, it is significant that SARS-CoV-2 spike glycoprotein molecules (“spikes”) can be found in serum either affixed to virions or free floating, detached from virions. Although in typical SARS-CoV-2 viral replication, spike protein is synthesized and then attached to newly formed virions inside the infected host cell, the unexpected leakage of spike protein outside that infected cell has been documented in vitro and clinically [171,172]. Additionally, it has been estimated that in SARS-CoV-2 viral replication, 10^5^ new virions emerge from an infected cell [173], typically grouped into infectious units each consisting of 10^4^ virions [173,174], with up to 65 attached spikes on each virion [142,143,144,145].

The endothelial cell may play an important role both as a focus of virally induced vascular damage caused by a SARS-CoV-2 infection and as a source of leaked spike protein during viral replication. Replicated SARS-CoV-2 virus from an infected alveolar cell could penetrate a virally compromised basement membrane joining the alveolar/capillary basal surfaces and then infect an endothelial cell [135,175]. Detached spike protein from an infected alveolar cell could likewise penetrate through an adjoining capillary wall. Alternatively, a SARS-CoV-2 virion in serum could attach to an endothelial cell, with the glycan-dense NTDs of viral spikes attaching to the heavily sialylated endothelial cell surface [125]. Such a virion snagged onto an endothelial cell would come into constant contact with a stream of RBCs, each flowing through a pulmonary septal capillary, flattening against the capillary’s somewhat smaller cross-sectional diameter walls [116,117,118].

With these RBCs rapidly flowing over the spikes on that snagged virion, subject to strong RBC-spike glycan bindings, it could become dislodged from that endothelial cell, possibly ripping off a piece of the endothelial cell membrane. If that endothelial cell had become virally infected, such a membrane rupture could cause leakage of replicated spike protein. These scenarios entailing damage to endothelial cells and associated leakage of spike protein into blood could account for the high levels of circulating endothelial cells (CECs) and holes in endothelial cells approximately the size of a SARS-CoV-2 viral capsid (the viral envelope) found in COVID-19 patients [114]. These scenarios would also be consistent with the endothelial damage [15,18,19,126,127,128] and associated spike protein traces on endothelial cells [129,130,131,132,133,134,135] that have been widely observed in such patients. 

Consider a hypothetical scenario in which one in a thousand of the endothelial cells of a COVID-19 patient were infected, damaged but not dislodged, and in which each released a full measure of unattached spike protein as a result of an altered or aborted viral replication cycle. That would represent 500-fold the number of endothelial cells that became so severely damaged as to be detected as CECs, and this scenario would yield 270 spike protein molecules per RBC circulating in blood (see Appendix A.1). Although Lam et al., as cited, found SARS-CoV-2 viral spike protein on 41% of RBCs from COVID-19 patients [36], those RBCs were examined after repeated washings, and it is possible that only a fraction of spike-studded RBCs were detected, with many others ensnared into clumps that evaded detection. In any case, a many-to-one ratio of spikes to RBCs could develop for some period of time in the vicinity of an infected body organ.

The highest estimate of SARS-CoV-2 serum viral load is approximately 100 billion virions per COVID-19 patient [173,176], at most one virion per 200 RBCs. It is therefore reasonable to consider that free-floating spike protein, detached from virions, e.g., as released per atypical viral replication per the in vitro and clinical studies noted [171,172], accounts for much of that which attaches to RBCs in COVID-19 patients. Regardless of whether a spike were virion attached or free floating, it would form a strong, multivalent attachment at its N-terminal end to an RBC. These bonds would extend from the spike’s NTD, having eight N-glycosylation sites, which for a betacoronavirus is indeed the typical point of initial attachment to a host cell [44,81,82,83,84,85,86]. From the NTDs on the spike’s three monomers, multiple bonds could form to GPA strands extending 5 nm from the RBC plasma membrane, spaced an average of 14 nm apart on the RBC surface [75], or to sialoglycoproteins of other types such as glycophorin B that are interspersed [177].

As noted above, spike protein glycans and GPA have the same species of SA (Neu5Ac) and of other terminal monosaccharides. Additionally, GPA [166] and NTD both have nanomolar spacings of glycans, and GPA has no known physiological function except as a decoy for pathogens [31,72,73,75]. It is thus reasonable that strong, multivalent attachments would form between spikes at their N-terminal ends and RBCs. These attachments could possibly be strong enough to dislodge a virally-attached spike when subject to forces of separation during blood flow.

Once a spike became attached to an RBC at its NTD on its N-terminal end, its C-terminal end could attach to another RBC through a bond formed from the N-glycan at N1194, the glycan closest to its C-terminal end. That N1194 glycan is the most sialylated of all 22 N-glycans, having four terminal SA monosaccharides [140,178], and could attach to a GPA strand extending 5 nm from an RBC membrane given that this N1194 attachment point is a lesser distance, approximately 4 nm, from the C-terminal end of the spike [140,179]. Additionally, glycans swing flexibly at their N-glycosylation attachment sites, and spike protein has two flexible “hinge” points between N1194 and its C-terminal end [140], further facilitating attachment from N1194 to GPA. Although the trimeric configuration of spike protein extends to its C-terminal (stalk) end, with three N1194 glycans thus capable of binding to GPA, the three monomers converge within 5 nm of each other at that end, and it is unclear if multivalent bonds of sufficient collective strength could form to attach the spike at that C-terminal end to a second RBC to maintain any durability during blood flow.

### Completing the Spike-Mediated Linkage of Two RBCs with IgG Antibody Targeting Spike RBD

Given two adjacent RBCs, each with a SARS-CoV-2 spike attached at the spike’s NTD, these two spikes in turn could be joined to each other by a molecule of IgG or another class of antibody that targeted viral spike RBD, with RBD being the most common antigen target of both natural and vaccine-generated antibodies against SARS-CoV-2 [180,181]. In an IgG antibody, Y-shaped, the ends of the two antigen-binding fragments are spaced approximately 15 nm apart [182], while the centers of the NTD and RBD domains of SARS-CoV-2 spike protein S1 are spaced approximately 5 nm apart on each monomer [84,85]. Therefore, such a complex of two spikes attached at their NTDs to RBCs, joined to each other by an IgG antibody at their RBDs, would space the RBCs approximately 15–20 nm apart, greater the 8 nm minimum separation imposed by forces of electrostatic repulsion between two RBC surfaces [168,170]. The binding affinities between SARS-CoV-2 spike protein and antibodies against it are generally strong, in the range of −10 to −15 kcal/mol [181]. 

Hemagglutination, as induced by whole SARS-CoV-2 virions and antibody, was proposed by Roe [29], while two groups of investigators experimentally demonstrated hemagglutination using a system closely related to that of the combination of RBCs, SARS-CoV-2 spike protein and antibodies as proposed here. Instead of spike protein, however, each of these two group constructed a fusion protein that linked spike protein RBD to an antibody fragment targeting the RBC surface. Using a classic assay technique that visibly detected hemagglutination, both groups obtained positive results when mixing human RBCs with constructed fusion proteins as described and anti-RBD antibodies of several types, including those from the sera of COVID-19 patients, using nanomolar concentrations of both fusion protein and antibody [183,184]. 

Were antibody required to induce hemagglutination in a COVID-19 patient, then this could not occur prior to its presence in serum. Yet in the typical course of COVID-19, serum antibodies would appear prior to significant deterioration of oxygenation saturation. In three studies using sensitive detection methods, antibodies against SARS-CoV-2 RBD or against spike S1 were observed in serum of most COVID-19 patients within a week of the onset of symptoms [185,186,187]. The most pronounced reductions in SpO2 in COVID-19 patients, however, typically occur at least a week following onset of symptoms [188,189]. Of course, some patients could experience breathing dysfunction early in the disease course, prior to the generation of antibodies, from causes other than hemagglutination. Indeed, as will be considered below, for 34 severe COVID-19 patients treated with a clinical agent indicated to be a competitive inhibitor of binding to spike protein, all but three had mean SpO2 normalizations of 55% within 12−24 h [190]. However, afterwards, patients typically required several more days for full recovery, consistent with additional causes of lung damage beyond rapidly reversible hemagglutination.

## 4. Testing for Hemagglutination Caused by SARS-CoV-2 Spike and for Inhibition by Competitive Binding

The hemagglutination assay, dating back to the 1940s [38,49,50,51], is a simple procedure still used widely, e.g., by blood banks to detect blood groups [183]. Human or animal RBCs in phosphate buffer solution are mixed with a hemagglutinating agent such as an influenza or betacoronavirus virus, typically in one well of a microwell plate, with the formation of a visually detectable interlaced sheet of RBCs if hemagglutination occurs [40,57,58,59]. This assay can be readily performed using trimeric SARS-CoV-2 spike protein mixed with human RBCs, with anti-spike RBD antibody added as well if needed. Al though hemagglutination has been demonstrated in vitro for SARS-CoV-2 virus using the hemadsorption assay, as noted [35], testing using spike protein rather than whole virus obviates the requirement for stringent safety protocols and allows the biochemistry to be explored at a more elemental level. This experiment is in line with past studies in which spike protein (not attached to virions) from two coronavirus strains was found to cause hemagglutination [191,192]. (See Appendix A.4.)

### Competitive Inhibition of SARS-CoV-2 Spike Protein Binding by Ivermectin (IVM)

If SARS-CoV-2 spike protein is found to induce hemagglutination, further insight into the underlying biochemistry and its clinical implications could be provided through the study of agents that inhibit spike-RBC attachments. Competitive binding to viral spike protein by such SA-rich agents as gangliosides [41] and fetuin [80], for example, has been studied. Toward the goal of identifying potential therapeutics for COVID-19, four molecular modeling studies collectively screened over 800 molecules for binding to SARS-CoV-2 viral spike protein [193,194,195,196]. The strongest or close to strongest binding affinity in each study was obtained for ivermectin (IVM), a macrocyclic lactone with multifaceted antiparasitic and antimicrobial activity, distributed in 3.7 billion doses for human diseases worldwide since 1987 [197,198,199]. Additional molecular modeling studies of binding to SARS-CoV-2 spike protein sites that focused on IVM in particular, including Lehrer and Rheinstein (2020) [200], likewise found strong binding affinities for IVM [201,202,203,204,205].

These findings are of interest given clinical, animal and epidemiological studies, including most of the 20 randomized clinical trials (RCTs) conducted to date, indicating efficacy of IVM against COVID-19 [197,206,207]. Yet interpretations of which of these RCTs for IVM treatment of COVID-19 are reliable have been controversial. One published study reporting no efficacy for IVM, for example, had switched IVM and placebo doses for 38 patients, systematically violated blinding, and shown distinctive signs of IVM use in the placebo group [208,209]. However, one preprint reporting efficacy of IVM was retracted [210]. IVM is suitable for mass use on a global scale, having been the mainstay of worldwide campaigns to eliminate two devastating scourges, Onchocerciasis and Lymphatic Filariasis [211]. It is safe even at much higher than the standard dose of 200 μg/kg [212,213], and the limited nature of its side effects were noted in the Nobel Committee’s 2015 award honoring its discovery and its record of improving the health and wellbeing of millions [214].

Certain in vivo findings suggest that the main activity of IVM against SARS-CoV-2 may apply to viral morbidity rather than infectivity, consistent with its main underlying biological mechanism being competitive inhibition of viral binding to host glycans. Two animal studies of IVM treatment at low human-equivalent doses, one for the SARS-CoV-2 virus in golden hamsters [215] and another for a related betacoronavirus (MHV-A59) in mice [216], found statistically significant treatment reductions in morbidities but either no reduction or a lesser reduction, respectively, in viral load. Some RCTs for IVM treatment of COVID-19 have found no reductions in viral load for most patients [217], or at most insignificant reductions in viral load accompanied by significant reductions in mortality [218] or symptoms [219] for the treatment groups. An RCT for prevention that tracked 42-day follow-up to a single 12 mg dose of IVM in 617 subjects found a 50% reduction in incidence of symptomatic COVID-19 (*p* = 0.003) and a 49% reduction in associated ARDS (*p* = 0.012) with respect to controls, but only a non-significant 8% reduction in relative incidence of positive PCR test results [220].

Supporting the above-cited indications of a reduction in viral morbidity being the notable benefit provided by IVM against COVID-19 were marked, short-term improvements in oxygenation saturation (SpO2) in two studies. Both studies tracked changes in SpO2 values in severe COVID-19 patients on room air before and within a day after treatment with the triple therapy of IVM, doxycycline and zinc. One of these studies found that in 34 severe COVID-19 patients with pretreatment SpO2 values ≤ 93, all but three had an increase in SpO2 within 12–24 h. These 34 patients had mean (±SD) SpO2 normalizations of 55.1% (±28.0%) at +12 h and 62.3% (±26.3%) at +24 h, with normalization defined as the percentage of increase in SpO2 with respect to that from pretreatment SpO2 to a fully normal SpO2 of 97 [190]. The second study found that in 19 COVID-19 patients with pre-treatment SpO2 values ≤ 90, SpO2 normalized by a mean of 65.2% within 24 h [221]. All patients in both these groups survived. Although IVM reaches peak plasma and tissue concentrations, respectively, approximately 4–8 h after oral administration [212,222,223], even if viral replication in lung tissue were frozen immediately after penetration with IVM, it would be difficult to explain such sharp increases in SpO2 within 12–24 h if they resulted from repair of damaged lung alveolar tissue.

A recent molecular modeling study computationally explored bindings of IVM to five SA-containing binding sites on SARS-CoV-2 spike protein NTD and to 17 other binding sites on NTD and RBD, calculating binding affinities as S-score values using AutoDock Vina software [224]. Most of the binding affinities of IVM to those five sialoside sites and to the other NTD and RBD sites were less than −7.0 kcal/mol (absolute values 7.0 kcal/mol). This study also found that computed binding affinities of IVM were less than −7.0 kcal/mol at five of 12 binding sites on CD147, suggesting that IVM could competitively inhibit viral bindings to SA-tipped glycoconjugate binding sites on host cells as well. For comparative reference, another study that explored physiologically relevant activity corresponding to computed Autodock binding values for a large set of HIV inhibitors and likely non-inhibitors found that a binding affinity less than −7.0 kcal/mol selected the HIV inhibitors with 98% sensitivity and 95% specificity [225]. The Lehrer and Rheinstein molecular modeling study found that IVM docked to one site on spike protein RBD with the very strong binding affinity of −18.05 kcal/mol [200].

Quantitative estimates of competitive inhibition by IVM for spike-mediated RBC aggregation are challenging to provide given complexities and unknowns concerning the binding of IVM with multiple sites on SARS-CoV-2 spike protein and possibilities for steric interference. The following chemical properties and parameters, however, are key. For a dose of IVM administered with food in the standard, non-aggressive range of 200–350 µg/kg, the peak plasma level (at +4 h) of IVM plus active metabolites would be approximately 412 nM, which amounts to 52,000 molecules of IVM and active metabolites per RBC in human blood (see Appendix A.3). Following an oral dose, 93% of IVM in blood will bind to plasma proteins, mainly albumin [226,227], with the bound fraction unable to penetrate capillary walls or cell membranes to impact tissue outside of the blood [228]. If IVM were found to bind to albumin at a different molecular region than that which binds to spike protein, then that IVM-albumin complex bound to spike would offer a significant degree of steric interference, with the albumin molecule spanning dimensions of 8 × 8 × 3 nm [229]. For IVM unbound to albumin, the rectangular dimensions spanning that molecule are 2 × 1 nm [230], which when bound to a site on spike protein would offer a lesser degree of steric interference.

A model of multivalent viral attachments to sialic acid binding sites on host cells that was predictive of experimental observations found that a competitive inhibitor of bindings to viral glycan sites having only moderate such binding affinities could significantly limit the collective strength of such attachments [98]. If spike-mediated RBC clumping required a secondary join of two spike RBD domains to each other by an antibody, then a bond of an IVM molecule to an RBD site with the very strong binding affinity of −18 kcal/mol as predicted [200] could by itself significantly inhibit such an attachment. Given the indicated dynamic nature of the viral spike-mediated formation of RBC rouleaux [120,121,122], even a moderate reduction in such collective binding strengths could shift the balance from spike-mediated RBC attachment to detachment under the turbulent forces of blood flow.

The scenario proposed above for rapid resolution of diminished oxygenation in COVID-19 patients by IVM through competitive binding to glycans on SARS-CoV-2 spike protein is of course hypothetical, yet subject to testing through the in vitro model proposed above. If SARS-CoV-2 trimeric spike protein were found to cause hemagglutination when mixed with RBCs, then this test could be extended to ascertain whether the addition of IVM at a physiological concentration would block hemagglutination, or whether the subsequent addition of IVM after spike protein and RBCs were previously mixed would reverse RBC clumping. Such reversal of hemagglutination indeed occurs with the viral generation of HE for HE-expressing viral strains such as common cold betacoronaviruses [38,58,60,61]. This same effect could be monitored clinically through microscopic examination of the blood of COVID-19 patients before and after administration of IVM, to check for presence and post-IVM absence of rouleaux. As noted, an agent that is active against SARS-COV-2 by binding to multiple spike protein sites would likely be more effective against assorted viral mutants than vaccine-generated antibodies targeting specific epitopes on RBD. This assumption, too, could be tested by performing this in vitro experiment using spike protein from different SARS-CoV-2 variants.

## 5. Discussion 

To put the findings discussed here for SARS-CoV-2 in historical context, many viruses use glycoconjugate receptors at host cell surfaces for initial attachment, and sialyl glycans were among the first viral receptors discovered [25,30,63,80,231]. Following observations in the 1940s of hemagglutination induced by the influenza virus [38,49,50,51,52,53,54,55,56] and in the 1950s of immune adherence by RBCs as a primal host defense mechanism against viruses and other pathogens [77,78], SA was identified in the 1970s as the key molecular group behind these viral–RBC attachments [232]. The SA-binding properties of several species of coronaviruses, including most of the human betacoronaviruses, were subsequently elucidated, as reviewed above, with those properties identified for SARS-CoV-2 through viral binding to an SA-coated nanoarray [94], the hemadsorption assay [35], and spike protein punctae found on 41% of RBCs from COVID-19 patients [36].

The specific arrangement and chemical composition of the glycans at the 22 N-linked glycosylation sites of SARS-CoV-2 protein have been closely studied, as summarized above. Yet the focus of many of these studies has been the role of these glycans, which swing flexibly at these attachment sites, in shielding spike epitopes from antibody recognition or in stabilizing the open and closed configurations of RBD as affects binding to ACE2 [84,140,163,164]. The active role of these glycans in attachment to host cells and hemagglutination is typically not considered. Here, we have reviewed that active role of glycan binding for viruses with the SA-binding properties of SARS-CoV-2. We have focused in particular on the possibility that for this virus, an overzealous primitive antiviral defense mounted by RBCs (having ubiquitous GPA surface molecules with no other known physiological function) may become counterproductive to the host, with RBC-viral attachments forming interlaced clumps that obstruct microvascular blood flow. Links between spikes to each other formed by anti-RBD antibodies could complete this chain of attachments between RBCs, compounding the collateral damage inflicted by the host’s immune defense. The rouleaux seen in the blood of COVID-19 patients could be a manifestation of this clumping caused by these interlaced viral–RBC bindings. As noted, the non-virulent character of the two of the five betacoronaviruses that express HE, an enzyme which releases viral–RBC bindings, provides further support for the proposed scenario.

Specifics of these glycan-mediated viral–RBC bindings have been considered here to further explore this hypothesis and lay the groundwork to experimentally test it. Useful to refine such testing is IVM, an agent that has been indicated to competitively bind to SARS-CoV-2 spike protein glycan sites, a biological mechanism that could explain the rapid increases in SpO2 obtained for hypoxic, severe COVID-19 patients after administration of IVM. Were IVM to reverse and/or inhibit RBC clumping induced by SARS-CoV-2 spike protein, this would further confirm the hypotheses presented here and sharpen indications of their clinical relevance. Such reversal of virally induced hemagglutination by IVM would also align with two other findings noted above: 93% binding of IVM to plasma proteins in blood, sharply limiting its penetration into extravascular tissue, and inhibition by IVM of morbidity, but not viral replication (which would occur primarily in extravascular tissue), in several clinical and animal studies.

It is important to appreciate, as noted earlier, that biological dynamics other than hemagglutination contribute to microvascular occlusion and that additional morbidities of COVID-19, including neurological dysfunction, are also of key concern. The CD147 transmembrane receptor, for example, is a key mediator of inflammatory response and a promoter of adhesion by RBCs, other blood cells and endothelial cells [22]. Additionally, the alpha-7 nicotinic acetylcholine receptor (α7nAChr), which is densely distributed on neuronal tissue, has been indicated in computational molecular docking studies to be a binding site for SARS-CoV-2 spike protein [233,234]. Such binding from spike protein to α7nAChr may contribute to the loss of smell and taste that is characteristic of COVID-19 infection, while α7nAChr also mediates an important anti-inflammatory pathway that could mitigate the cytokine storm, as reviewed [224]. A recent molecular modeling study has reported binding affinities of physiologically relevant strength of IVM to both CD147 and α7nAChr in addition to those to NTD and RBD sites on SARS-CoV-2 spike protein [224]. These additional findings, if validated in vitro and/or clinically, could further explain the observed clinical benefits of that agent for COVID-19.

## 6. Conclusions

Attachments of glycans on SARS-CoV-2 spike protein to RBCs and to other blood cells and endothelial cells may be central to the microvascular morbidities of COVID-19. An in vitro experiment is proposed to test these attachments, in particular the binding considered here between spike protein glycans and SA terminal residues of GPA surface molecules on RBCs, possibly with a further linkage provided by anti-RBD antibodies. If hemagglutination is found to occur when SARS-CoV-2 trimeric spike protein is mixed with RBCs, possibly with anti-RBD antibody required as well, further insight can be provided by testing the capability of the macrocyclic lactone, IVM, to block these attachments through competitive binding.

## Data Availability

Not applicable.

## Abbreviations

The following abbreviations are used in this manuscript:

α7nAChr	alpha-7 nicotinic acetylcholine receptor
CEC	circulating endothelial cell
COVID-19	coronavirus disease 2019
GPA	glycophorin A
HE	hemagglutinin esterase
IVM	ivermectin
Neu5Ac	α5-N-acetylneuraminic acid
NTD	N-terminal domain
RBC	red blood cell
RBD	receptor-binding domain
RCT	randomized clinical trial
SA	sialic acid

## Appendix A. Calculations and Notes on the Proposed Hemagglutination Experiment

### Appendix A.1. Calculation 1

Estimate of the number of spike protein molecules that could be released, unattached to virions (e.g., per these in vitro and clinical studies [171,172]), from host endothelial cells infected with the SARS-CoV-2 virus.

Given an estimated 10^5^ new SARS-CoV-2 virions that emerge from an infected cell [173] and up to 65 attached spikes on each virion [142],^143^,^144^,^145^), up to 6.5 × 10^6^ spikes per infected cell could be released. There are an average of five liters of blood [235], with an RBC count of ~4.8 × 10^9^/mL [236] (amounting to close to the total estimate of approximately 25 trillion RBCs [72]), and one trillion endothelial cells per human [237]. The mean number of circulating endothelial cells (CECs) in COVID-19 patients was found to be approximately 400 per ml of blood [114]. This yields 400 × 5000 = 2 × 10^6^ CECs per patient.

Consider the following scenario, in which for every CEC detected in a COVID-19 patient, presumably representing an endothelial cell subject to extreme viral damage that ripped it out of the capillary wall, 500 other endothelial cells were virally infected and sufficiently damaged such that they leaked a full load of 6.5 × 10^6^ spike protein molecules into blood, with the normal viral replication cycle being altered or aborted, but without being dislodged from the capillary wall. That would yield 2 × 10^6^ × 500 = 10^9^ damaged endothelial cells each releasing 6.5 × 10^6^ spike molecules, for a total of 6.5 × 10^15^ spike molecules released into blood.

With an average of 2.4 × 10^13^ RBCs in a human, the total number of spike molecules would represent approximately 270 spike protein molecules per RBC. With 10^12^ endothelial cells per human, the number of damaged but not dislocated endothelial cells considered in this scenario, 10^9^, would constitute one-thousandth of the total number of endothelial cells.

### Appendix A.2. Calculation 2

Concentration of SARS-CoV-2 trimeric spike protein required to yield a ratio of 1000 spike protein molecules to one RBC given a 10% in solution.

The concentration of RBCs in human blood is ~4.8 × 10^9^/mL [236].

Thus, for a 10% solution of RBCs, that figure is 4.8 × 10^8^/mL

The molecular mass of SARS-CoV-2 trimeric spike protein (aa 1273) is ~426 kDa [238].

1000 molecules of spike protein per RBC in 1 mL of 10% RBC solution requires a spike protein weight of:

1000 × 4.8 × 10^8^ divided by Avogadro’s number, multiplied by the molecular mass of spike protein = (4.8 × 10^11^/6.022 × 10^23^) × 426,000 g = 0.797 × 10^−13^ × 426,000 g = 3.40 × 10^−7^ g = 0.340 µg.

If a hemagglutination test is performed in, e.g., a 96 microwell plate, each microwell filled to 0.2 mL, that would then require 68 ng of spike protein per well to yield a ratio of 1000 spikes per RBC with a 10% RBC solution.

The molarity of spike protein solution for each such experiment would be:

(3.4 × 10^−7^ g/4.26 × 10^5^ moles/g) per ml = 0.798 × 10^−10^ moles per L = 0.0798 nM.

### Appendix A.3. Calculation 3

Peak plasma level after administration of a standard dose of IVM with a fatty meal.

The peak plasma level (C_max_) of IVM administered to fasting subjects (achieved 4 h after ingestion) for a dose range of 400–700 µg/kg, typical for COVID-19 treatment, is 96.2 ng/mL, or 109.9 nM [223]. With absorption of IVM 2.5-fold greater when taken with a high-fat meal v. when fasting, and with linear pharmacokinetics up to single doses of 120 mg [212], that would yield a C_max_ of 120.3 ng/mL or 137.4 nM for an IVM dose of 200–350 µg/kg administered with a fatty meal.

The active metabolites of IVM reach approximately double its C_max_ at approximately the same t_max_ [222,239]. So this yields an effective C_max_ of IVM plus active metabolites of 360.9 ng/mL, or 412.2 nM.

Given the concentration of RBCs in human blood of ~4.8×10^9^/mL [236], this yields 51,720 molecules of IVM and active metabolites per RBC in human blood at the peak plasma level of IVM for the dose stipulated.

### Appendix A.4. Notes on the Proposed Hemagglutination Experiment

Since the composition of glycans in recombinantly generated SARS-CoV-2 spike protein depends upon the cell type used for spike generation, that cell type must be human, e.g., HEK 293 embryonic kidney cells, and human RBCs must also be used. To obtain a 1000-to-1 ratio of trimeric spike protein molecules per RBC using a 10% solution of human RBCs in a microwell filled to 0.2 mL would require 68 ng of spike protein per microwell (see Appendix A.2 above). Although under clinical conditions, the ratio of spike protein molecules to RBCs would likely be less, characteristics of the in vitro environment might require a higher ratio of spikes to RBCs for hemagglutination to be readily detectable. As noted above, an antibody against SARS-CoV-2 spike protein, such as CR3022, a monoclonal IgG antibody targeting RBD, may also be needed in the mix, in quantities per studies cited above of 2–12.5 ng per microwell [183,184]. Note also that rouleaux can form either as simple cylindrical stacks of RBC disks or as more complex, branched clusters [240]. While the latter might manifest as hemagglutination upon visual inspection, simple stacks of RBCs might be detectable only with microscopic examination or viscosity measurements. In any case, even if visibly detectable hemagglutination did occur, those additional two forms of examination would provide useful additional insights as to the nature of this clumping.
